# Investigations concerning the impact of consumption of hot beverages on acute cytotoxic and genotoxic effects in oral mucosa cells

**DOI:** 10.1038/s41598-021-01995-9

**Published:** 2021-11-26

**Authors:** Benjamin Ernst, Tahereh Setayesh, Armen Nersesyan, Michael Kundi, Michael Fenech, Claudia Bolognesi, Miroslav Mišík, Masood Ghane, Seyed Fazlollah Mousavi, Siegfried Knasmüller

**Affiliations:** 1 Department of Medicine I, Institute of Cancer Research, Medical University of Vienna, Vienna, Austria; 2 Center for Public Health, Medical University of Vienna, Vienna, Austria; 3 School of Pharmacy and Medical Sciences, University of South Australia, Adelaide, Australia; 4 Centre of Healthy Ageing and Wellness, Faculty of Health Sciences, Universiti Kebangsaan Malaysia, 43650 Bangi, Selangor Malaysia; 5 Environmental Carcinogenesis Unit, Ospedale Policlinico San Martino, Genoa, Italy; 6 Department of Microbiology, Islamic Azad University of Tonekabon, Mazandaran, Iran; 7 Department of Microbiology, Pasteur Institute of Iran, Tehran, Iran

**Keywords:** Cytological techniques, Health care

## Abstract

Consumption of very hot beverages and foods increases the incidence of oral and esophageal cancer but the mechanisms are not known and the critical temperature is not well defined. We realized a study with exfoliated cells from the oral cavity of individuals (n = 73) that live in an area in Iran which has the highest incidence of EC worldwide. Consumption of beverages at very high temperatures is a characteristic feature of this population. We analyzed biomarkers which are (i) indicative for genetic instability (micronuclei that are formed as a consequence of chromosomal damage, nuclear buds which are a consequence of gene amplifications and binucleated cells which reflect mitotic disturbances), (ii) markers that reflect cytotoxic effects (condensed chromatin, karyorrhectic, karyolitic and pyknotic cells), (iii) furthermore, we determined the number of basal cells which is indicative for the regenerative capacity of the buccal mucosa. The impact of the drinking temperature on the frequencies of these parameters was monitored with thermometers. We found no evidence for induction of genetic damage but an increase of the cytotoxic effects with the temperature was evident. This effect was paralleled by an increase of the cell division rate of the mucosa which was observed when the temperature exceeded 60 °C. Our findings indicate that cancer in the upper digestive tract in drinkers of very hot beverages is not caused by damage of the genetic material but by an increase of the cell division rate as a consequence of cytotoxic effects which take place at temperatures over 60 °C. It is known from earlier experiments with rodents that increased cell divisions lead to tumor promotion in the esophagus. Our findings provide a mechanistic explanation and indicate that increased cancer risks can be expected when the drinking temperature of beverages exceeds 60 °C.

## Introduction

Esopharyngeal cancer and oral cancer are widespread forms of cancer. Approximately 670,000 new cases of esopharyngeal and oral cancers, resulting in more than 300,000 deaths are recorded worldwide per year^1,2^. Despite efforts to improve the treatment, esopharyngeal cancer still has a poor prognosis (i.e. the 5 year survival rate is in the range between 10 and 18%^3^). It is known that several lifestyle and nutritional factors increase the risks (see for example^4,5^). Studies concerning the impact of the temperature of foods and beverages indicated that it is an important factor for squamous cell carcinomas in the oesophagus and in the oral cavity of population groups in certain areas of the world for example in South American countries and in certain regions of the Iran^6–9^. The evidence for the associations led to the classification of consumption of hot beverages as a Group 2A carcinogen (“probably carcinogenic to humans”) by the International Association for Research on Cancer^10^. A very high prevalence of esophageal cancer (EC) is observed in Brazil, Uruguay and Paraguay, where hot mate tea is consumed frequently^5,7,11^; another “hotspot” are Northern Provinces of Iran at the Caspian sea where the incidence of EC reaches the highest levels in the world^12,13^. The consumption of hot (black and green) tea is typical for this region and probably the most important risk factor^13^. Apart from beverages, also intake of hot foods may play an important role, but only few studies have been published, which address this issue^6,7,14^.

The cellular mechanisms by which heat causes formation of malignant cells are at present unclear; also the critical temperature is not well defined as most cancer studies are based on self-reported temperatures. This question was addressed in the present study. Most investigations concerning the impact of hyperthermia on cellular alterations which lead to formation of cancer cells, were performed under in vitro conditions with pro- and eukaryotic cells (for details see “Discussion” section). Several modes of action may play a role, namely damage of the genetic material^15–18^ as well as acute cell death leading to increased proliferation^19–21^.

This is the first study in which the impact of consumption of hot beverages on cellular alterations (acute cytotoxicity—genetic damage—mitotic activity) was monitored. Furthermore, it is the first study in which the crucial temperature which causes nuclear alterations was defined. The results of these investigations made it possible to draw conclusions about which cellular mechanisms are related to malignant transformation of cells of upper digestive tract altered by consumption of hot beverages.

**Figure 1:**
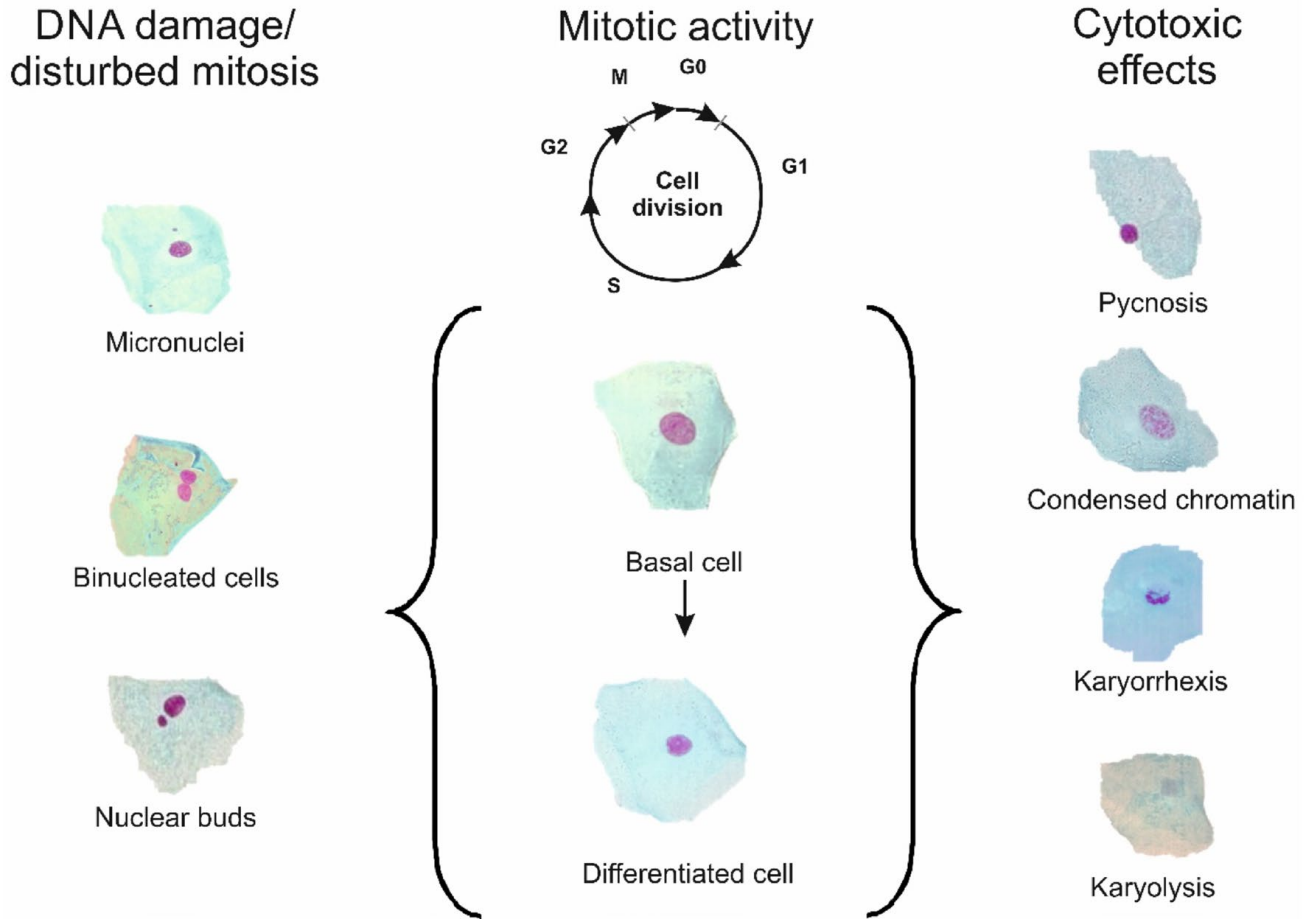
Morphology of different anomalies which are scored in MN-cytome experiments with buccal cells. For a detailed description of the characteristics of the different anomalies and scoring criteria see Refs.^23,50^.

We performed a micronucleus (MN) cytome study with buccal cells of 73 participants from a high risk region in Northern Iran and analysed the impact of the temperature and of the amount of tea and coffee consumption on various parameters which reflect the stability of the DNA, cell proliferation and acute cytotoxic effects in exfoliated cells of the oral mucosa. Micronuclei (MN) are extra-nuclear DNA-containing bodies which are formed as a consequence of malsegregation of chromosome fragments or whole chromosomes during mitosis as a consequence of damage to chromosomes and/or the mitotic apparatus^22^. Nuclear buds (Nbuds) reflect extrusion of excess nuclear DNA due to gene-amplification events, while binucleated cells (BN) are a consequence of disturbed cell division resulting in cytokinesis failure^23^. Endpoints which reflect acute cytotoxicity include karyorrhexis (KR), karyolysis (KL), cells with condensed chromatin (CC) and pyknosis (PY); the number of basal cells is an indicator of the mitotic activity and regenerative capacity of the oral mucosa (for details see Refs.^23,24^). The different forms of nuclear anomalies, which were scored in this study, are shown in Fig. 1. It is well documented that buccal MN-cytome assay biomarkers reflect increased cancer risks caused by lifestyle factors (tobacco-, betel- and khat-chewing) and occupational exposures to genotoxins (for example see Refs.^25,26^). In 2009, a standardized/validated protocol was published by Thomas et al.^23^ and clear scoring criteria were defined. The present study was performed in agreement with these guidelines.

## Results

Results for endpoints reflecting instability of the genetic material (the number of cells with MN and the total number of MN/cell and Nbuds) and failure of cell division (BN cells) are listed in Table 1. It is evident that the temperature had no impact on the frequencies of these anomalies. Table 2 shows the effects of hot tea consumption on parameters reflecting acute cytotoxic effects and cell proliferation (basal cells). We found a highly significant increase of all these anomalies. The most pronounced effect was the drastic raise of the number of pycnotic and karyolytic cells. These frequencies were in participants who drank extremely hot beverage substantially higher than those found in “normal” drinkers (temperatures ˂ 55 °C). Also, the rates of KR cells and of cells with CC increased with the drinking temperature; the increase of these anomalies in participants consuming beverages at temperature ≥ 70 °C compared to those drinking them at moderate temperatures was in the range between 2.75- and 2.78-fold, respectively.

**Figure 2:**
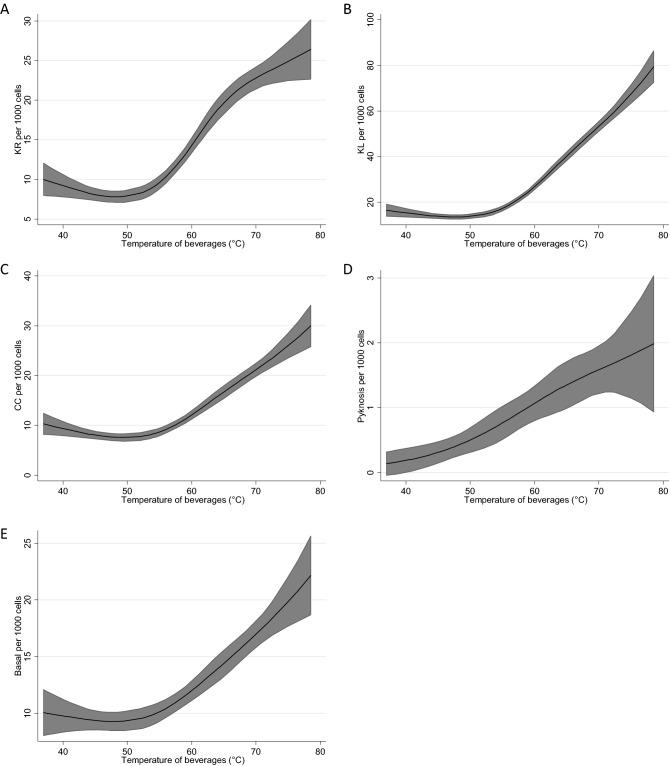
(**A**–**E**) Estimated number (and 95% confidence intervals) of cellular anomalies and basal cells as a function of the average temperature of beverages. Estimation by Poisson regression with restricted cubic splines (*KR* karyorrhexis, *KL* karyolysis, *CC* condensed chromatin).

Except for pyknosis (an anomaly which is rare and for which only the linear component was significant) all nuclear anomalies indicating cell damage and the number of basal cells increased only at temperatures ≥ 55 °C. These effects became pronounced at 60 °C and above; the increases are linear for all anomalies, except for KR; in this case we found a slight curvature if 65 °C were exceeded (Fig. 2A–E).

Tables 1 and 2 summarize the impact of the drinking temperature, the number of cups/day and of different demographic factors on OR-values and corresponding CI and p-values. Acute cytotoxicity parameters (KR, KL, CC, PY) as well as the number of basal cells increased with the drinking temperature. The number of drinks had no impact on the formation of nuclear anomalies in general. The lack of an effect may be due to the narrow range (i.e. the participants consumed between 3 and 5 cups/day). The age of the participants was associated with a small (but significant) increase of KL cells but had no impact on the formation of other anomalies. Also, the BMI values did not affect the outcome of the analysis.

**Table 1:** Impact of the drinking temperature, the numbers of drinks/day and of demographic factors (age, BMI) on induction of nuclear anomalies reflecting DNA damage and disturbance of cell division in buccal cells. p-values from Poisson regression.

Endpoint	Predictor	OR	95% CI	p-value	p for trend
MN cells	Tea temperature < 55 °C	1 (Ref)			
	55–59 °C	1.19	0.73–1.94	0.487	
	60–64 °C	1.17	0.70–1.94	0.553	
	65–69 °C	0.94	0.50–1.77	0.854	
	70 + °C	1.16	0.76–1.77	0.487	0.738
	> 60 °C	1.00	0.74–1.35	0.979	
	Overweight (BMI 25–29.9)	0.99	0.71–1.40	0.971	
	Age (per decade)	0.95	0.79–1.15	0.611	
	# drinks/day	0.96	0.76–1.23	0.773	
Total MN	Tea temperature < 55 °C	1 (Ref)			
	55–59 °C	1.20	0.75–1.90	0.448	
	60–64 °C	1.11	0.69–1.78	0.677	
	65–69 °C	1.23	0.71–2.11	0.461	
	70 + °C	1.24	0.83–1.83	0.290	0.385
	> 60 °C	1.08	0.82–1.43	0.577	
	Overweight (BMI 25–29.9)	1.00	0.73–1.38	0.993	
	Age (per decade)	0.93	0.78–1.11	0.405	
	# drinks/day	1.00	0.80–1.26	0.986	
Nuclear buds	Tea temperature < 55 °C	1 (Ref)			
	55–59 °C	1.03	0.74–1.43	0.882	
	60–64 °C	1.02	0.73–1.42	0.910	
	65–69 °C	1.26	0.88–1.81	0.210	
	70 + °C	1.25	0.95–1.63	0.106	0.146
	> 60 °C	1.11	0.92–1.34	0.275	
	Overweight (BMI 25–29.9)	1.06	0.85–1.32	0.622	
	Age (per decade)	0.99	0.87–1.11	0.823	
	# drinks/day	0.96	0.82–1.13	0.638	
BN	Tea temperature < 55 °C	1 (Ref)			
	55–59 °C	1.05	0.87–1.26	0.632	
	60–64 °C	0.92	0.76–1.12	0.412	
	65–69 °C	0.92	0.73–1.16	0.477	
	70 + °C	1.01	0.86–1.18	0.944	0.653
	> 60 °C	0.94	0.84–1.05	0.262	
	Overweight (BMI 25–29.9)	0.95	0.83–1.08	0.435	
	Age (per decade)	0.92	0.86–0.99	0.026	
	# drinks/day	0.96	0.87–1.05	0.340	

*MN* Micronuclei, *BN* binucleated cells, *OR* Odds ratio, *CI* confidence interval.

Number of participants per temperature group: < 55 °C (n = 21); 55–59 °C (n = 11); 60–64 °C (n = 15); 65–69 °C (n = 6); 70 + °C (n = 20).

**Table 2:** Impact of the drinking temperature, the number of drinks/day and of demographic factors (age, BMI) on induction of nuclear anomalies reflecting acute cytotoxicity and cell proliferation (basal cells) in buccal cells. p-values from Poisson regression.

Endpoint	Predictor	OR	95% CI	p-value	p for trend
KR	Tea temperature < 55 °C	1 (Ref)			
	55–59 °C	0.99	0.81–1.20	0.916	
	60–64 °C	1.80	1.51–2.14	< 0.001	
	65–69 °C	2.33	1.95–2.78	< 0.001	
	70 + °C	2.75	2.40–3.16	< 0.001	< 0.001
	> 60 °C	2.20	2.01–2.42	0.000	
	Overweight (BMI 25–29.9)	1.04	0.94–1.16	0.409	
	Age (per decade)	1.00	0.95–1.06	0.888	
	# drinks/day	0.85	0.79–0.91	< 0.001	
KL	Tea temperature < 55 °C	1 (Ref)			
	55–59 °C	0.95	0.82–1.10	0.477	
	60–64 °C	1.81	1.60–2.06	< 0.001	
	65–69 °C	2.40	2.11–2.74	< 0.001	
	70 + °C	3.86	3.50–4.26	< 0.001	< 0.001
	> 60 °C	2.84	2.66–3.04	< 0.001	
	Overweight (BMI 25–29.9)	0.98	0.91–1.05	0.522	
	Age (per decade)	1.13	1.09–1.18	< 0.001	
	# drinks/day	0.94	0.89–0.99	0.014	
CC	Tea temperature < 55 °C	1 (Ref)			
	55–59 °C	1.06	0.87–1.29	0.557	
	60–64 °C	1.25	1.04–1.50	0.018	
	65–69 °C	1.72	1.42–2.09	< 0.001	
	70 + °C	2.78	2.42–3.19	< 0.001	< 0.001
	> 60 °C	2.15	1.95–2.37	< 0.001	
	Overweight (BMI 25–29.9)	1.07	0.96–1.19	0.231	
	Age (per decade)	1.04	0.98–1.10	0.211	
	# drinks/day	1.02	0.94–1.10	0.637	
PY	Tea temperature < 55 °C	1 (Ref)			
	55–59 °C	1.37	0.64–2.91	0.417	
	60–64 °C	1.83	0.89–3.74	0.098	
	65–69 °C	3.06	1.51–6.19	0.002	
	70 + °C	3.83	2.19–6.70	< 0.001	< 0.001
	> 60 °C	2.71	1.86–3.93	< 0.001	
	Overweight (BMI 25–29.9)	1.00	0.67–1.48	0.991	
	Age (per decade)	0.98	0.79–1.23	0.882	
	# drinks/day	0.90	0.68–1.20	0.479	
Basal	Tea temperature < 55 °C	1 (Ref)			
	55–59 °C	0.95	0.79–1.14	0.597	
	60–64 °C	1.12	0.94–1.33	0.219	
	65–69 °C	1.52	1.26–1.83	< 0.001	
	70 + °C	1.83	1.60–2.09	< 0.001	< 0.001
	> 60 °C	1.51	1.37–1.66	< 0.001	
	Overweight (BMI 25–29.9)	0.96	0.86–1.07	0.459	
	Age (per decade)	1.05	0.99–1.11	0.121	
	# drinks/day	1.02	0.94–1.10	0.620	

*KR* Karyorrhexis, *KL* karyolysis, *CC* condensed chromatin, *PY* pyknosis, *OR* odds ratio, *CI* confidence interval.

Number of participants per temperature group: < 55 °C (n = 21); 55–59 °C (n = 11); 60–64 °C (n = 15); 65–69 °C (n = 6); 70 + °C (n = 20).

## Discussion

The results of the present study which was performed with exfoliated cells from the upper digestive tract (oral mucosa) are relevant for the elucidation of the cellular mechanisms by which consumption of hot beverages and foods causes EC and enable the assessment of the critical temperature leading to adverse effects.

It has been repeatedly reported that inhabitants that live in the vicinity of the sampling area (in the Golestan province) have increased prevalence of EC^7,13,27^. It was postulated in these studies that high temperature of beverages plays an important role as a risk factor. This is not only true for cancer incidence in Iran but also for other areas of the world^6,10^. Furthermore, it was also shown earlier that high temperature of foods and beverages lead to alterations which are causally related to cancer; results of an endoscopic study of precancerous lesions in the high risk area in Northern Iran showed increased rates of esophagitis and esophageal squamous dysplasia which represent precarcinogenic lesions for EC^28^. These changes were clearly associated with consumption of hot beverages. However, it was also found that consumption of deep fried foods, ethnicity and age have an impact on the incidence of EC^29^.

As described in the results section, we did not detect an increase of the frequencies of MN, indicating that hot tea consumption does not cause chromosomal damage. These findings are unexpected as earlier in vitro studies reported increased levels of chromosomal aberrations and MN rates when mammalian cells were cultured at higher temperatures (see for example^30,31^); furthermore also higher levels were found in combination experiments with radiation^32^. The indicator cells were grown in these experiments continuously at temperatures ≤ 40 °C; therefore, the exposure conditions were quite different compared to those in consumers of hot beverages where the maximal temperatures reach 70 °C and cells are exposed for a relatively short time. It was postulated that high temperatures lead to impairment of DNA-repair processes and DNA-damage^33–35^; in this context it is notable that Kampinga and Laszlo^33^ criticized a Japanese study^36^ which postulated that heat induced cell killing is caused by double strand breaks (DSBs). Our results underline the validity of this criticism and show that pronounced cytotoxicity is observed in mucosa cells already in absence of chromosomal damage (which results from DSBs).

We found that the consumption of hot tea has a strong impact on the viability of the cells; i.e. the rates of different nuclear anomalies which are characteristic for acute cytotoxicity increased with the temperature (see Figs. 2A–D). As described in detail in the results section, the rate of pycnotic cells, which are characterized by condensed dead nuclei, was substantially higher in individuals that drank beverages at temperatures ≥ 70 °C; also the numbers of other anomalies (KR, KL and CC) were clearly enhanced, but the effects were less pronounced. The increase in basal cells with the drinking temperature may have been caused by increased mitotic division probably as a consequence of the induced cytotoxic effects causing loss of surface mucosal cells and may play an important role in the formation of cancer cells. It is well known that increased cell division is a hallmark of human cancer^37,38^. In this context it is notable that Rapozo et al.^20^ published an interesting study with mice which were treated orally with dimethylnitrosamine and hot water. The authors found an increase of the mitotic activity of basal cells and in parallel hyper-proliferation of premalignant lesions. The same observations were also made in a number of further studies with other initiating carcinogens^39–41^. The increase of the proliferation rates may be triggered by activation of heat-shock proteins which are induced by high temperatures that are involved in several cell signalling pathways controlling cell division and apoptosis (for details see Ref.^19^). It is notable that only healthy participants took part in the present study. It can be not excluded that genetic damage is involved in later stages of the development of cancer caused by consumption of hot beverages as it is well documented that increased mitotic activity can lead to replication errors and as consequence result in damage of the genetic material^42,43^. Furthermore, there is some evidence that several other factors such as age, ethnicity and food carcinogens may play an additional role in the initiation of esophageal cancer in Northern Iran^44^.

The results of our study enabled the assessment of the critical temperature, which causes cell death and induces proliferation. In regard to anomalies which reflect acute toxic effects, the critical temperatures are ≥ 60 °C. Also the mitotic activity increases significantly when the temperature of the beverages exceeds 60 °C. In this context it is notable that the authors of a prospective Iranian cancer study found that hazard ratio values for EC increased by ca. 40% in individuals who drank tea at temperatures ≥ 60 °C^27^. This is the only study in which the drinking temperature of the beverages was measured. In all other investigations information of the drinking temperature was assessed with questionnaires; in the most recent one the participants consumed water at 65 °C and were asked if they drink tea at higher or lower temperatures^45^. In animal studies, tumor-promoting effects in the oropharyngeal tissue were found with temperatures between 65 and 70 °C which increased the rates of preneoplastic lesions, while no effects were seen with temperatures between 55 and 60 °C^20,40^.

Taken together, the present results suggest that the induction of EC and OC by consumption of hot beverages may result from increased cell proliferation as a consequence of induced acute cytotoxic effects. This effect was seen after consumption of beverages with temperature ≥ 60 °C in healthy subjects and it is obviously the first step of malignant transformation of the cells in upper digestive tract. However, it cannot be excluded that genetic damage occurred at later stages of cancer development. It is known that hot drinks cause esophagitis and dysplasia which represent premalignant lesions^28^, and it is well documented that inflammations are associated with an increase of chromosomal aberrations that lead to formation of MN^46^.

## Materials and methods

### Study participants

**Figure 3:**
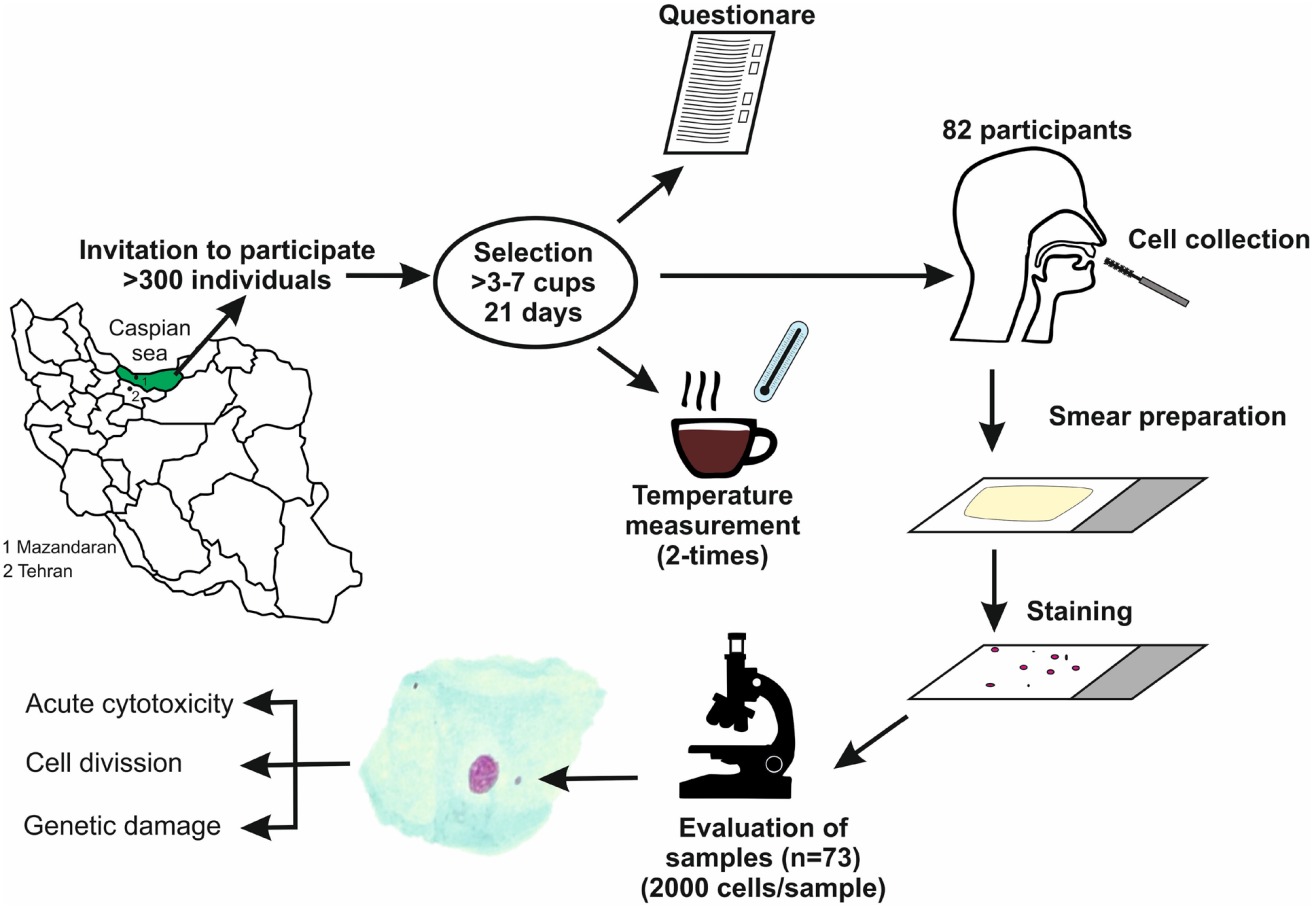
Schematic representation of the study design. More than 200 individuals were asked if they are willing to participate in the study; only those who were healthy non-smokers and consumed ≥ 3 cups of hot beverage daily over a period of 21 days were eligible. The participants filled in questionnaires concerning their lifestyle and demographic data. The temperature of the consumed beverages was measured twice with thermometers (at the start and end of consumption period). Mouth cells were collected with cytobrushes, fixed and stained and evaluated microscopically. We sampled in total 82 individuals, only slides from 73 subjects contained a sufficient number of cells for cytome analyses.

The experimental design of the study is illustrated in Fig. 3. This study was realised with 73 inhabitants of Nowshar (Mazandaran Province, Islamic Republic of Iran). Initially, cells were collected from 82 subjects. Nine samples were excluded; two because of microbial contaminations, in seven samples the number of cells was insufficient.

Demographic data were collected with questionnaires and are listed in Table 3. All participants were healthy male volunteers who were occupationally not exposed to known mutagenic carcinogens. Exclusion criteria were chronic use of non-alcoholic mouth rinses (which may cause nuclear alterations^47^), dental X-ray examination ≤ 1 month before the sampling, tobacco chewing and smoking, intake of vitamin supplements and food additives, betel quid chewing, oral and systemic diseases and use of pharmaceuticals. The study group comprised students, school teachers, shop assistants, waiters and office workers. Potential participants were asked first about the amount of daily tea and coffee consumption. Individuals who consumed 3–4 cups per day were considered eligible. All participants consumed the beverages at least 3 weeks before sampling because it is known that the turnover rate in buccal cells is between 7 and 21 days^23,44^. Their health status and the demographic characteristics were recorded with questionnaires (24% reported “excellent”, 35% “very good” and 41% “good” health). Age, body mass index (BMI) and life style habits (number of hot drinks per day, diet, and physical activity) were recorded. We also asked the participants to provide detailed information about consumption of tea and other hot beverages. Most of them drank black tea and coffee (for details see Table 3). The beverages are consumed in the study area in small glasses; the amounts per cup varied between 130 and 150 ml. All participants were omnivorous, non-smokers and alcohol abstinent (for details see Table 3).

**Table 3:** Demographic factors and drinking habits of the participants.

Number of participants	73
Age (year)	33.6 ± 8.6
Range	21–50
Quantity of hot drinks per day (cups)	3.8 ± 1.2
Range	3–7
Initial temperature of drinks (°C)	59.9 ± 10.8
Range	39–80
Final temperature of drinks (°C)	55.4 ± 10.3
Range	36–76
Body mass index (kg/m^2^)	25.6 ± 1.6
Range	22.1–27.9
Number of cells scored per participant	1778 ± 276.5
Range	1005–2035
Types of hot drinks consumed	N = 22 only black tea N = 5 only green tea N = 46 black tea and coffee

### Ethical approval

The study protocol was approved by the Islamic Azad University-Tonekabon Branch, Iran (registration number IR.IAU.TON.REC.1399.095) in accordance with the Declaration of Helsinki. Written informed consent was obtained from all patients prior to enrolment.

### Measurements of the temperature of the beverages

To determine the drinking temperature, each participant was invited to consume a cup of black or green tea (130 ml) which was served hot. The temperature of the beverage was measured twice, namely at the start of the consumption period and shortly before the end (when ca. 80% of the beverage had been consumed). Measurements were performed with an infrared thermometer (non-contact digital laser temperature gun “Helect”, Shenzhen JEWY Tech Co. Ltd, Shenzhen, China). The values used for the statistical calculations were the averages of these two values.

### Collection of exfoliated cells and cytogenetic analyses

Exfoliated buccal cells were collected as described earlier^48,49^ from the posterior part of the oral cavity close to the esophagus. Immediately before sampling, the participants were asked to rinse their mouth 2–3 times with tap water. Subsequently, the cells were collected by use of cytobrushes (Heinz Herenz, Hamburg, Germany) and were smeared on microscopic slides with a few drops of distilled water. From each participant, two slides were prepared. After 24 h, they were fixed with 80% cold methanol, then they were placed in 80 ml glass beakers with 5.0 M HCl at room temperature for 30 min, rinsed with distilled water for 5 min and stained subsequently with Schiff’s reagent (Sigma-Aldrich, Steinheim, Germany) for 90 min, washed with running water for 5 min and then counterstained with 0.2% (w/v) Light Green (Sigma-Aldrich, Steinheim, Germany) for 30 s. From each participant, 1500–2000 buccal cells were evaluated. Nuclear anomalies were scored in differentiated and basal cells as suggested by Thomas et al.^23^ under bright light (400×); when MN were detected they were confirmed under fluorescent light (1000× with oil immersion (Nikon Microphot-FXA, Tokyo, Japan). The nuclear anomalies were recorded according to the criteria defined by Thomas et al.^23^ and Bolognesi et al.^50^. The slides were evaluated by an experienced scorer and cross-checked by a second experienced scorer.

### Statistical methods

Data were evaluated by Generalized Linear Models using Poisson deviates with a log link. The main independent variable of interest was the temperature of the beverages categorized into 5 predefined ranges: < 55 °C, 55–59 °C, 60–64 °C, 65–69 °C, ≥ 70 °C. Furthermore, a restricted cubic spline regression was performed with 4 knots at the percentiles recommended by Harrell^51^. The non-linear terms were highly significant in all nuclear anomalies reflecting cytotoxic effects except pyknosis and in basal cells. In all analyses age, BMI, and number of hot beverages consumed per day were included as potential confounders.

The study had a 90% statistical power to detect a 30% increased odds ratio for most nuclear anomalies at the 5% level of significance. All analyses were performed by Stata 13.1 (StataCorp, College Station, TX, USA).

## Abbreviations

BN: Binucleated
CC: Condensed chromatin
EC: Esophageal cancer
KL: Karyolysis
KR: Karyorrhexis
MN: Micronucleus
Nbud: Nuclear bud
OC: Oral cancer
PY: Pyknosis
